# Population diversity control based differential evolution algorithm using fuzzy system for noisy multi-objective optimization problems

**DOI:** 10.1038/s41598-024-68436-1

**Published:** 2024-08-01

**Authors:** Brindha Subburaj, J. Uma Maheswari, S. P. Syed Ibrahim, Muthu Subash Kavitha

**Affiliations:** 1 School of Computer Science and Engineering, Vellore Institute of Technology, Chennai, India; 2 School of Information and Data Sciences, Nagasaki University, Nagasaki, Japan

**Keywords:** Multiobjective optimization, Differential evolution, Local search, Fuzzy systems, Engineering, Machine learning

## Abstract

The objective measurements of the real-world optimization problems are mostly subject to noise which occurs due to several reasons like human measurement or environmental factors. The performance of the optimization algorithm gets affected if the effect of noise is higher than the negligible limit. The previous noise handling optimization algorithms use a large population size or multiple sampling at same region which increases the total count of function evaluations, and few methods work for a particular problem type. To address the above challenges, a Differential Evolution based Noise handling Optimization algorithm (NDE) to solve and optimize noisy bi-objective optimization problems is proposed. NDE is a Differential Evolution (DE) based optimization algorithm where the strategies for trial vector generation and the control parameters of DE algorithm are self-adapted using fuzzy inference system to improve the population diversity along the evolution process. In NDE, explicit averaging based method for denoising is used when the noise level is higher than negligible limit. Extending noise handling method enhances the performance of the optimization algorithm in solving real world optimization problems. To improve the convergence characteristics of the proposed algorithm, a restricted local search procedure is proposed. The performance of NDE algorithm is experimented using DTLZ and WFG problems, which are benchmark bi-objective optimization problems. The obtained results are compared with other SOTA algorithm using modified Inverted Generational Distance and Hypervolume performance metrics, from which it is confirmed that the proposed NDE algorithm is better in solving noisy bi-objective problems when compared to the other methods. To further strengthen the claim, statistical tests are conducted using the Wilcoxon and Friedman rank tests, and the proposed NDE algorithm shows significance over the other algorithms rejecting the null hypothesis.

## Introduction

Many real-world problems are formulated as optimization problems. While modeling the optimization problems, noise may arise from different roots and such problems are called noisy optimization problems. There are various sources from which noise may arise such as, measurement errors, incompleteness in data and environmental factors, due to which it is difficult to attain appropriate objective function value^1–3^. The impact of such noise factors in optimization problems can be, different objective value results over multiple evaluations for a same individual. The other impact of noise is, when the noise strength is high, population will contain large number of poor solutions, due to which the environment selection process is affected and deviates the search direction away from the true front^4^. Thus, when the effect of noise is high it may influence more in search process^5^. It is vital to consider noise factor while modelling the optimization problem. Including the noise factor along with objective function value while evaluation is presented in Eq. 1.

1 $$F\left(x\right)+\varepsilon$$

where $\varepsilon$ is the noise factor, $\varepsilon \sim N(0,{\sigma }^{2},I)$. The standard deviation component ${\sigma }^{2}$ denotes the noise strength and $I$ is the identity matrix.

Evolutionary algorithms (EAs) mimic the natural selection and genetic inheritance principles. EA samples a population of candidate solutions, and new solutions are generated through selection, mutation and crossover operations. Promising solutions are selected for the next generation. EAs are robust in handling noisy optimization problems. The performance of EA may deteriorate when level of noise is high. Few notable draw backs of EA include fine tuning the control parameters associated with the algorithm according to the problem and premature convergence.

Real-world Engineering optimization problems mostly requires optimization of multiple objectives simultaneously. These problems are classified as multi-objective optimization problems (MOPs). Mathematical representation of a MOP is given by Eq. 2,

2 $$Minimize/Maximize F(Y)= [{F}_{1}\left(Y\right), {F}_{2}\left(Y\right),..,{F}_{m}\left(Y\right)]$$

where, $Y=[{Y}_{1}, {Y}_{2},.., {Y}_{d}]$ is a vector of $d$ decision variables subject to boundary constraints and $F(Y)$ is the objective vector with $m$ objective functions. The objective of multi-objective optimization problem is to attain a set of pareto optimal solutions that exhibits good convergence and diversity characteristics.

Evolutionary algorithms are one among the widely used methods to solve such multi-objective optimization problems for the past two decades, since it is simple and much prior knowledge is not required and has a greater potential to perform global search. Nondominated sorting genetic algorithm II (NSGA-II)^6^, Strength pareto evolutionary algorithm (SPEA2)^7^ are popular elitism based multi-objective evolutionary algorithms. As stated earlier, the performance of EA can be affected if the noise level is high.

The existing techniques designed for dealing with such optimization problems with noise factor can be categorized in to averaging, ranking and modeling based methods. Where, the averaging based methods can be categorized as explicit and implicit averaging methods. Explicit averaging method evaluates a solution for multiple times, and the average value is considered as the objective function value. In the implicit averaging model, size of the population is increased for reducing the impact of noise. Ranking based optimization methods are grouped in to probability ranking and clustering based ranking. In the probabilistic ranking method, the process of selecting fitter individuals is modified. Instead of the conventional dominance relation based selection operator, the probabilistic dominance factor is used to mitigate the effect of noise which estimates the dominance probability amongst two solutions. The clustering ranking method of noise handling is based on estimation of clustering radius in order to select fitter solutions. In the modeling based method of handling noisy optimization problems, a model is derived based on a solution set where the impact of noise could be less while compared to its effect on a single solution.

Given such wide range of algorithms to handle noisy optimization problems, the limitations associated with each of the methods are also to be considered. In general, averaging based methods are computationally expensive, since the function evaluations count it takes is high. In ranking based optimization methods, the results may be inaccurate since the dominance based ranking is replaced with probabilistic or other alternate techniques. Modeling method based algorithms may not be applicable to a wide range of optimization problems and can be suitable in solving problem with specific characteristics.

From the above study, we propose a Differential Evolution based noise handling optimization algorithm (NDE) to optimize noisy bi-objective optimization problems. The optimizer used is FAMDE-DC^8^ as it is robust and efficient in handling optimization problems of varied characteristics. The main contributions include:

- Differential evolution based noise handling optimization algorithm (NDE) is developed using the FAMDE-DC algorithm as the base optimizer combined with noise handling method.
- Adaptive switching technique^9^ is extended through which denoising method is applied only when the noise factor is high. Explicit averaging based method of denoising is used for handling noise.
- To improve the exploitation properties of the NDE algorithm, a restricted local search technique is proposed.
- Experimenting the performance of NDE algorithm. Benchmark optimization problems from DTLZ and WFG suite are used with noise inclusion. Modified inverted generational distance and the hypervolume indicators are utilized for performance evaluation. Results are compared with other algorithms and further non-parametric statistical test is conducted to strengthen the findings.

The paper is organized as follows, the related work is discussed in section “Related works”, the FAMDE-DC optimizer and the technique to measure the noise strength is given under section “Preliminaries”. In section “proposed algorithm”, the proposed NDE algorithm is detailed. In section “experimentation”, the experimental setup, test problems, performance metrics, results are presented. The conclusions are given in section “conclusions”. The notations used in the research and its expansion are listed in Table 1.

**Table 1:** Notations.

$\varepsilon$	Noise
${\sigma }^{2}$	Noise strength
$I$	Identity matrix
$CR$	Crossover rate
$NP$	Population size
$F$	Scaling Factor
$LE$	Learning period
$popdiversity$	Population diversity
$refdiversity$	Reference diversity
$CRm$	Crossover rate mean
${\lambda }_{res}$	Resampling ratio
${N}_{re}$	count of solutions to be resampled
${f}_{pro}$	probabilistic based rounding function
$d$	Problem dimension
$s$	Noise strength
$\theta$	Acceptance threshold
${LS}_{Int}$	Local search interval
${LS}_{Feval}$	Local search number of function evaluations

## Related works

Differential Evolution algorithm^10^ is simple to extend, robust single objective optimization algorithms. Several algorithms by extending DE to solve MOPs are presented by various researchers. In^11^, authors introduced DE algorithm based on pareto dominance approach suitable to optimize multi-objective problems and the algorithm is named as Paret-frontier differential evolution algorithm (PDE). In Differential evolution for multi-objective optimization (DEMO)^12^, the base DE algorithm is combined with pareto ranking and crowding distance based sorting methods suitable to be applicable to optimize multi-objective optimization problems. The DEMO algorithm is further extended by^13^ to solve many objective optimization problems by using correlation-based ordering of the objectives for the selection of conflicting objectives subset and they named it as $\propto$-DEMO. In^14^, the authors have presented a multi-objective self-adaptive differential evolution (MOSADE) in which the non-dominated solutions are retained using external elitist archive and further diversity is improved using crowding entropy measure. Differential evolution based multi-objective optimization by controlling the population diversity through self- adaptation of strategies used for trial vector generation and the control parameters using fuzzy system is achieved by FAMDE-DC algorithm^8^. In^15^, authors have proposed a self-adaptive Trajectory optimization method used to optimize the problem associated in the UAV based mobile edge computing system. Differential Evolution algorithm is improved using a distance indicator and two stage mutation strategy suitable to optimize multimodal multi-objective problems^16^. The above works show the importance and potential of the algorithms in solving optimization problems.

Some of the existing multi-objective evolutionary algorithms developed to handle noisy optimization problems are summarized. In^16^, authors have used iterative resampling procedure to lower the effect of noise. They have adapted a varying number of samples for a solution depending on the current noise factor in the search space. Two approaches are used for uncertainty reduction, such as resampling and increasing population size^17^ which is applied to solve optimization problem in feedback control of combustion. Explicit averaging and modeling based method of denoising^9^ is used to reduce the noise and the authors have proposed adaptive switch strategy to choose the noise treatment and type based on effect of noise. The population size is increased or decreased based on the observed objective function values in the predefined number of iterations^18^. Population size control based evolutionary strategy named pcCMSA-ES^19^, where linear regression and hypothesis test are used to detect noise effect, upon which the population size is varied. The learning rate parameter is adapted based up on the noise ratio^20^, the empirical results of this learning rate adaptation when compared to resampling or increasing the size of samples is better but, the convergence rate is not optimal.

To reduce noise, the authors have used stochastic and significance based dominance methods for solution selection^21^. Probability based ranking is used for selecting the fitter solutions to handle noise^22^. Clustering based ranking scheme^23^ is used to handle noisy optimization problems. Algorithm based on restricted Boltzmann machine is used to build the probabilistic model and is hybridized with particle swarm algorithm to handle noisy optimization problems^24^. Regularity model is combined with NSGA-II algorithm for denoising^25^. Adaptive switch strategy^9^ is introduced through switch and select amongst the denoising techniques such as averaging and modeling methods based on the noise strength measurements. In^26^, authors have used radial basis function networks as denoising method.

In Filters based NSGA-II (FNSGA-II)^27^, mean and wiener filters are included with optimization algorithms to handle noise in images and signals. These filters help to reduce the noise factor balancing convergence and diversity. A noise handling method for surrogate assisted evolutionary algorithms^28^ by using radial basis function is proposed. Further, sampling strategies are chosen based on the convergence and diversity characteristics using adaptive switch technique. A Gaussian and Regularity model based NSGA-II algorithm named GMRM-NSGA-II is suggested to handle noisy multi-objective optimization problems. Population is divided to subpopulations and the above two models are applied to each of the population to improve convergence and diversity^30^.

The summary of literature review is given in Table 2.

**Table 2:** Literature review summary.

Refs.	Algorithm name	Technique	Test problem	Performance metric	Performance analysis
^9^	TSEA	1.Two stage evolutionary algorithm 2. Adaptive switching based on noise level 3. output solution selection to choose fitter non-dominated solutions	DTLZ and WFG problems	Modified inverted generational distance and Hypervolume	1.Optimiztion performance is good for unimodal and multi-modal problems 2. not effective in solving WFG problems higher level of noise
^28^	MS-MOEA	1.Adaptive model switch-based surrogate assisted evolutionary algorithm SAEA (MSMOEA) 2. IBEA $\in$+ is the basic optimizer 3. Switches adaptively between Radial basis function network and gaussian regression for noise reduction	DTLZ 1 to DTLZ7 test problems (Two and three objectives)	Inverted Generational distance	1.Best results for 12 problems 2.additive noise is studied, multiplicative noise experimentation not done
^30^	GMRM-NSGA-II	1. Gaussian model and regularity model based on fast elitist non-dominated sorting genetic algorithm 2. population split into subpopulation, and extends different model and selection techniques on each population	ZDT, DTLZ and WFG	Inverted Generational Distance	1.Performs better in handling problems with varied properties like modality, inseparable, etc 2. increased time consumption is a limitation
^27^	FNSGA-II	1. Fliters based NSGA-II 2. mean and weiner filters are applied for denoising 3. To handle noise in image, signals	ZDT, DTLZ and WFG	Inverted Generational distance	1. Acheieved good results for most of the problems 2. Performance limitations observed for problems, where True front is discontinuous
^39^	RTEA	1. Rolling tide evolutionary algorithm 2. elite archive with regular resampling of solutions in it 3. refinement phase during later stages of evolution to improve accuracy	CEC2009 test problems	Noise misinformation measure, Inverse generational distance, Hypervolume	1.Optimization performance is better for most problems compared to other algorithms 2. Limitations is, adaptation of resampling according to convergence stage will be beneficial
^25^	RM-NSGA-II	1. Regularity model in NSGA-II 2. regularity model is included like a reproduction operator 3. Extra denoising to choose appropriate sample points	ZDT, DTLZ and WFG	Generational Distance, Minimal spacing, Inverted Generational Distance	1.Improved performance of NSGA-II based algorithm in handling noisy optimization problem 2.Limitation observed in solving multimodal problem

## Preliminaries

The base optimizer FAMDE-DC and the technique to measure the noise strength is presented in this section.

### FAMDE-DC algorithm

FAMDE-DC^8^ is a multi-objective optimization algorithm. It is a Differential Evolution (DE) based algorithm. Crossover rate $(CR)$ value, which is a vital control parameter is self-adapted by using fuzzy system in order to improve the diversity among population. A pool of strategies for trial vector generation^29^ is used to generate trial vectors and the strategies are self-adapted based on their success index in previous generations, which indicates how successful a strategy is in generating solutions which are entering in to successive generation after selection process. The selection of solutions that are fitter is performed through fast non-dominated sorting^6^, improved with controlled elitism^30^ and dynamic crowding distance techniques^31^. Algorithm 1 shows the steps in FAMDE-DC algorithm and is detailed below.

**Algorithm 1:**
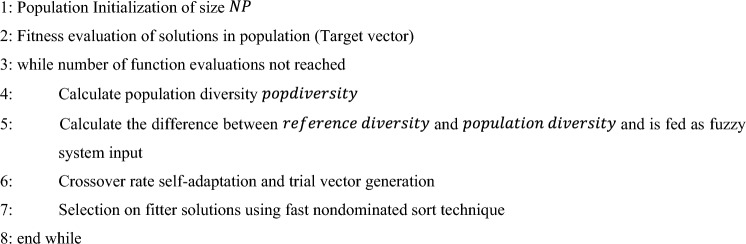
FAMDE-DC.

#### Initialization

The initial population set of size ($$NP$$) is generated randomly covering the search space boundary limits. Next the control parameters $$CR$$ and $$F$$ of the DE algorithm are initialized. The learning period ($$LE)$$ is set as 50.

#### Fitness evaluation

Objective function value is estimated for all the solutions in the population set.

#### Crossover rate adaptation

The control parameter crossover rate is adapted using fuzzy inference system to control the diversity of population. After the fitness function evaluation, the current generation population diversity $(popdiversity)$ estimated using distance to average point technique^32^. Reference diversity variable $\left(refdiversity\right)$ is set at 0.15. The difference between the current population diversity and reference diversity is estimated and given as input parameter to the single input/output fuzzy inference system. The fuzzy system maps the input with the implication rule set generated and it outputs the changes required to be made in the crossover rate in order to improve the population diversity. This change factor is used in crossover rate value generation, thus the crossover rate is self-adapted.

#### Trial vector generation

There are various strategies widely used in DE based algorithms for trial vector generation. The performance of the strategies varies among the optimization problems to which it is applied. Their performance characteristics even differs during the various stages of evolution, thus choosing a strategy for solving a particular optimization problem based on trial and error method is time consuming. Thus, a strategy pool with four trial vector generation strategies is extended^29^. During the initial learning period $(LE)$ the strategies are chosen with equal probability and later the strategies are chosen based on its success index value. The success index is an estimation of the percentage of solutions generated using a particular strategy, successfully chosen for the next generation after the selection process. The control parameter value crossover rate $(CR)$ is generated through normal distribution $N(Mean,Std)$, where the crossover rate mean $(CRm)$ is initially set at 0.5 and later is adapted using the population diversity and success index. The value of standard deviation set at 0.1. The scaling factor $(F)$ is generated using the normal distribution with mean and standard deviation values 0.5 and 0.3 respectively.

#### Selection

The trial and the target vectors are combined in to population size of $2NP$ solutions. Fast non-dominated sorting^6^ technique is used to split the solutions in to front levels based on non-domination factor. Solutions are selected based on controlled elitism^30^ and dynamic crowding distance^31^ techniques which ensures solutions from all the fronts are selected in order to improve the lateral diversity of the pareto front.

### Measuring noise strength

Resampling is a common method used to measure and quantify the strength of the noise. The steps involved in estimation of noise strength is given below^9,33^. The very first step in measuring noise strength involves, estimating the count of resampling solutions which are utilized to observe noise strength which is estimated using the formula in Eq. 3.

3 $$\begin{aligned} N_{res} = & \;f_{pro} \left( {NP*\lambda_{res} } \right), \\ f_{pro} \left( x \right) = & \;\left\{ {\begin{array}{*{20}c} {\left\lfloor {x + 1} \right\rfloor } & {based on probability\;x - \left\lfloor x \right\rfloor } \\ {\left\lfloor x \right\rfloor } & {otherwise} \\ \end{array} } \right. \\ \end{aligned}$$

where, the variable $NP$ is the size of the population set, the resampling ratio is given by ${\lambda }_{res}$ and this parameter limits the percentage of solutions that are to be re-evaluated. The count of solutions to be resampled is given by ${N}_{res}$ and ${f}_{pro}$ is the probabilistic based rounding function.

After estimating the count of resampling solutions, the second step is to choose randomly the ${N}_{res}$ number of solutions to be resampled. The third step is resampling the solutions and the technique of resampling differs based on the usage of explicit and implicit averaging denoising method. Upon resampling we will be left with two sets. For instance, let the solution set with noise inclusion is ${x}_{i}{\prime}={x}_{i}+N(0,{\sigma }^{2})$, where $i=\{\text{1,2},..d\}$ and $d$ represents the problem dimension. The sets before and after resampling are: ${\mathcal{L}}_{1}=\{{\widetilde{f}}_{1}, {\widetilde{f}}_{2},\dots ,{\widetilde{f}}_{{N}_{re}},{\widetilde{f}}_{{N}_{re}+1},\dots {\widetilde{f}}_{N}\}$ and ${\mathcal{L}}_{2}=\{{\widetilde{f}}_{1}{\prime}, {\widetilde{f}}_{2}{\prime},\dots ,{\widetilde{f}}_{{N}_{re}}{\prime},{\widetilde{f}}_{{N}_{re}+1},\dots {\widetilde{f}}_{N}\}$.

The fourth step is calculation of rank change, for each of the solutions that are resampled and is given in Eq. 4.

4 $${\Delta }_{i}=rank\left({\widetilde{f}}_{i}{\prime}\right)-rank\left({\widetilde{f}}_{i}\right)-\text{sign}(rank\left({\widetilde{f}}_{i}{\prime}\right)-rank\left({\widetilde{f}}_{i}\right))$$

where, the variable $rank\left({\widetilde{f}}_{i}\right)$ and $rank\left({\widetilde{f}}_{i}{\prime}\right)$ is the rank of solution in the set $\mathcal{L}$. The $\text{sign}()$ function returns + 1/-1/0 based on the whether argument is positive/negative/otherwise, this function aids in eliminating the change of rank created by the resampling solution. The rank change fall within the range: $\left|{\Delta }_{i}\right| \in \{\text{0,1},2,..2N-2\}$.

The last step is measuring the noise strength $s$ and is given in Eq. 5.

5 $$s=\frac{1}{{N}_{re}}\sum_{i=1}^{{N}_{re}}(2\left|{\Delta }_{i}\right|-{\Delta }_{\theta }^{lim}\left(rank\left({\widetilde{f}}_{i}{\prime}\right)-{1}_{{\widetilde{f}}_{i}{\prime}>{\widetilde{f}}_{i}}\right)-{\Delta }_{\theta }^{lim}\left(rank\left({\widetilde{f}}_{i}\right)-{1}_{{\widetilde{f}}_{i}>{\widetilde{f}}_{i}{\prime}}\right))$$

where, in the above equation rank change ${\Delta }_{i}$ is compared with variable ${\Delta }_{\theta }^{lim}$ which is limit based on the acceptance threshold $\theta$, and it indicates $\theta /2th$ percentile in the sequence of possible change of rank ${\mathbb{S}}=\{\left|1-R\right|,\left|2-R\right|, \dots ,|2N-1-R|\}$. The steps involved include generation of the ${\mathbb{S}}$ sequence, next the sequence ${\mathbb{S}}\mathbb{^{\prime}}$ is obtained by arranging ${\mathbb{S}}$ and at last $\theta /2th$ percentile is calculated from sequence ${\mathbb{S}}\mathbb{^{\prime}}$. $1$ is the indicator function and if its argument is matched correct then + 1 is returned else 0 is returned.

Thus, the strength of the noise given by variable $s$ gives the range of noise in the objective function. If the result $s<0$ then noise is within the limit of acceptance else, the resampled solutions have contributed to the change in rank beyond the acceptable limits and noise has huge effect over objective function.

## Proposed algorithm

**Figure 1:**
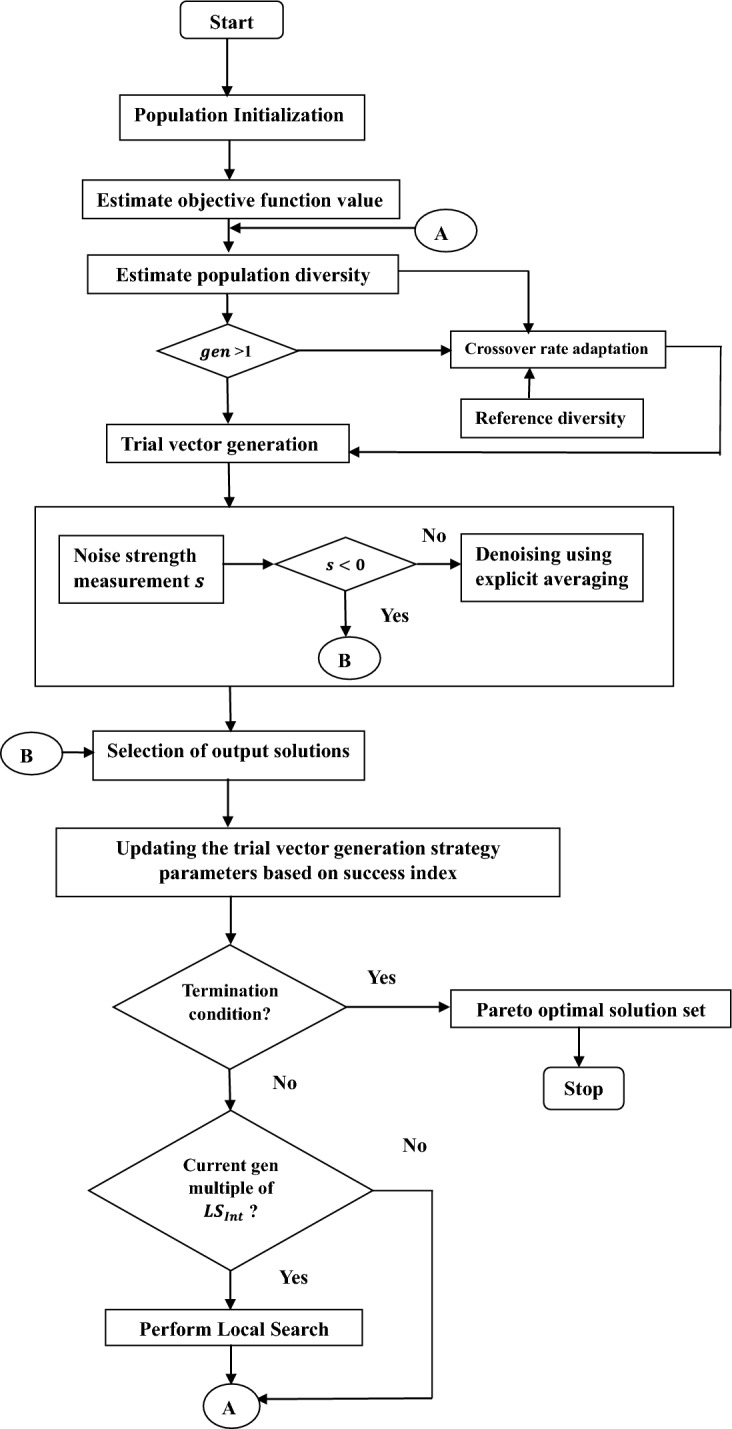
Flowchart representingthe proposedNDE algorithm.

The proposed Differential evolution based noise handling optimization algorithm (NDE) is detailed in this section. The algorithm and flowchart are given below in Algorithm 2 and Fig. 1 respectively.

**Algorithm 2:**
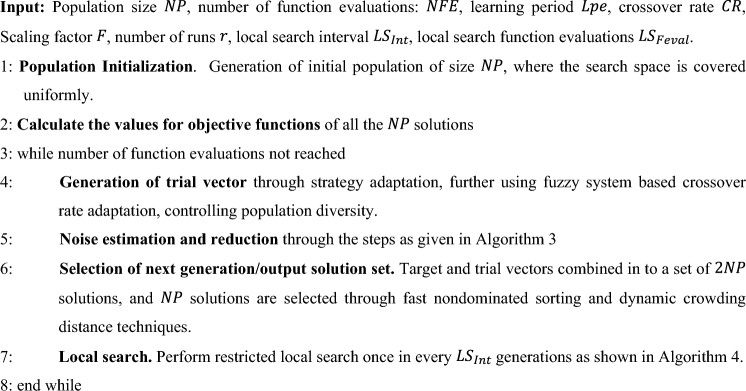
NDE.

### Initialization

Initial population set of size $NP$ is generated randomly.

### Trial vector generation

Trial vector is generated using a pool of strategies for trial vector generation^8,29^ as used in the base optimizer FAMDE-DC. The strategies are adapted based on success index and crossover rate is adapted using fuzzy system.

### Noise estimation and reduction

The effect or strength of noise is estimated through techniques as discussed in section “Measuring noise strength” with following modifications suitable to be applicable multi-objective optimization problems. The trial vector of $NP$ solutions are evaluated with fast non-dominated sorting technique^6^ and categorized in to non-dominated sets or fronts based on the domination count. The fitness of each solution is thus estimated using the fast non-dominated sorting based technique. The solutions used to measure the strength of noise for resampling is selected randomly from first front and, if sufficient solutions are not available in first front, then solutions from subsequent fronts are chosen till the required ${N}_{re}$ solutions are selected. If the estimated noise strength given by variable $s$ is less than 0, then the effect of noise is minimal and it is not required to denoise. Else, the noise is to be reduced using denoising method. The process for noise strength measurement and reduction is given in Algorithm 3.

**Algorithm 3:**
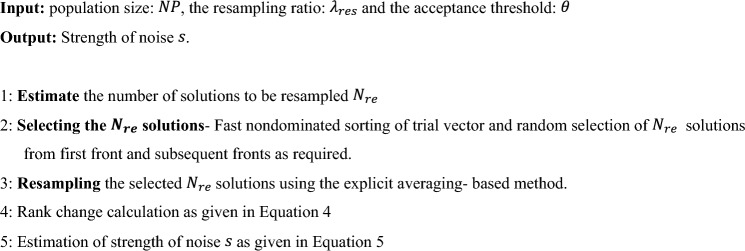
Noise strength measurement and reduction.

Explicit averaging-based method for denoising is applied to reduce strength of noise. This method uses resampling technique to denoise the values of objective functions for the selected solutions. In general, the resampling technique is applied for all the solutions for a specified count and averages the attained values as value for objective function. Selecting a solution set and resampling solutions in this set has shown significant improvement in the algorithm’s performance in denoising, and function evaluations utilized for resampling is thus wisely used through this method^9^.

In the proposed NDE algorithm, set of solutions are selected for resampling and each solution in the set are resampled twice. Fast non-dominated sorting is applied to the trial vector and solutions are categorized as fronts. The solutions listed in first front are set of non-dominated solutions. The solutions for resampling are selected from the first front. During the early stage of evolution, the number of solutions in the first front will be less and at later stage of evolution, the non-dominated solutions in the first front will be more. Thus, the solutions for resampling are chosen from the non-dominated solution set by forming a hyper box^9^ using the ideal point $({pop}_{min})$ and nadir point $({pop}_{max})$. These ideal and nadir points are chosen as in Eq. 6.

6 $$\begin{gathered} pop_{i} = \min \left\{ {f_{i} \left( {x^{j} } \right)\left| {j = 1, 2, \ldots , R} \right.} \right\} \in pop_{min} , \hfill \\ pop_{i} = \max \left\{ {f_{i} \left( {x^{j} } \right)\left| {j = 1, 2, \ldots , R} \right.} \right\} \in pop_{max} \hfill \\ \end{gathered}$$

$\forall i\in (\text{1,2},\dots ,m)$, where $m$ indicates number of objectives, $R$ is the count of non-dominated solutions in first front retrieved after performing non-dominated sorting. The solutions inside the hyper box are chosen for resampling.

### Selection

To select solutions for next generation, target and the trial vectors are combined to form a set of $2NP$ solutions, where $NP$ is the size of the population. This combined population set is subject to fast non-dominated sorting. The required $NP$ solutions are selected from first front, which is the set of non-dominated solutions. If sufficient number of solutions are not available in first front, then solutions from subsequent fronts are used. If more than required number of solutions are available in a front then, Dynamic Crowding Distance (DCD)^30^ technique is used to select the required solutions from the front. DCD ensures maintaining uniform diversity across the selected solutions.

### Local search

The function evaluations consumed during the explicit averaging-based method of denoising may reduce the convergence properties of the algorithm. Moreover, the crossover rate $CR$ self-adaptation using fuzzy system through population diversity control may also affect the convergence. Thus, to improve the convergence, exploitation of solutions in promising search region is done using a restricted local search algorithm. Local search based optimization method searches for a locally better solution compared to the current solution chosen for exploitation in its proximity, and if one such solution is found it is added to the population which improves the convergence rate.

In the present NDE algorithm, we propose a restricted local search technique. The local search is performed in regular prespecified intervals ${LS}_{Int}$ and for a limited number of function evaluations ${LS}_{Feval}$, through which exploitation at larger rate is restricted. The current population best solution is chosen from the first front (the set of non-dominated solutions). Search is performed in the proximity of chosen solution for ${LS}_{Feval}$ times and if a solution better (dominates) than the current population best solution or better than any solution in first front is identified through the search, then the identified solution replaces a random solution in the population. Thus, this restricted local search procedure, applied once in every ${LS}_{Int}$ generation tries to find a better solution through exploitation and improve the convergence rate. The ${LS}_{Int}$ and ${LS}_{Feval}$ values are selected based on trial and error basis and best parameter values are selected for further experimentation. The restricted local search method is given in Algorithm 4.

**Algorithm 4:**
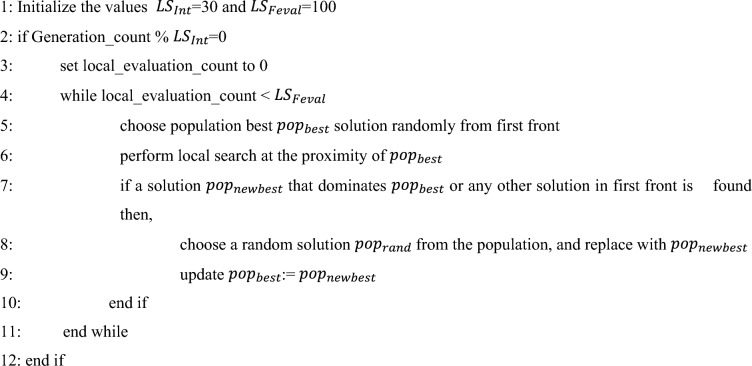
Restricted local search.

## Experimentation

To evaluate the performance of the proposed NDE algorithm, DTLZ^34^and WFG^35^ test problems are taken. The above test problem set are multi-objective unconstrained optimization problems and varying noise levels (0.1, 0.2, 0.5) is used for experimentation, and is detailed in section "Test problems". The performance metrics used for experimentation are modified inverted generational distance (IGD^+^)^36^ and hypervolume (HV)^37^ , which helps to assess both convergence and diversity properties of the attained solution set and the metrics are detailed in section "Performance metrics".

The performance of proposed NDE algorithm is compared with five multi-objective evolutionary algorithms namely, two stage evolutionary algorithm (TSEA)^9^, two archive algorithm (Two_Arch2)^38^, regularity model in NSGA-II (RM-NSGA-II)^25^, Noise-tolerant Strength Pareto Evolutionary Algorithm (NTSPEA)^2^, rolling tide evolutionary algorithm (RTEA)^39^ and Filters based NSGA-II (FNSGA-II)^27^. Except Two_Arch2 all other algorithms are developed to solve noisy optimization problems. The initialization of values for the various parameters used in proposed NDE algorithm are listed in Table 3.

**Table 3:** Parameters and initial values.

Parameter	Value
Number of function evaluations	50,000
Number of decision variables	DTLZ test problems- 30
	WFG test problems (WFG1, WFG4-WFG9)-30
	WFG2, WFG3- 31
Number of independent runs/trials	30
Population size	100
Initial crossover rate generation- mean and standard deviation values	$CR(\text{0.5,0.1})$
Scaling factor generation- mean and standard deviation values	$F(\text{0.5,0.3})$
Reference diversity ($refdiv$)	0.20
Learning period ($LE$) in generations	50
Local search interval count (${LS}_{Int}$)	30
Local search number of function evaluations (${LS}_{Feval}$)	300
Resampling count	2
Acceptance threshold $\theta$	0.2
Resampling ratio ${\lambda }_{re}$	0.95

### Test problems

Sixteen test problems are used to evaluate the performance of the proposed algorithm. All the problems are multi-objective unconstrained optimization problems. From DTLZ test suite^34^ seven problems (DTLZ1 to DTLZ7) are taken and from WFG test suite^35^ nine problems (WFG1 to WFG9) are taken. The number of decision variables and objectives can be scaled up to required numbers. These sixteen problems exhibits different properties (concave, multimodal, biased, etc.) make it suitable to be used to test the performance of the proposed optimization algorithm. Three objective problems are considered for investigation.

The properties of these problems are listed in Table 4. The significance of using above problems in evaluating optimization algorithms is the availability of true pareto optimal front. Further, in DTLZ problems the number of objectives is scalable, test problems DTLZ5 and DTLZ6 have degenerated pareto fronts and distance functions are added in all the DTLZ test problems. WFG Problems include complex characteristics like many problems are non-separable, we may also observe few problems are deceptive, problems where pareto fronts are mixed shape. The availability of problems with such complex characteristics and availability true optimal fronts for the benchmark problems like above, significantly contributes in conducting the experimentation in the optimization field.

**Table 4:** Test problems and its properties.

Test problem	Geometry	Modality	Separable/non-separable	Biased
DTLZ1	Linear	Multi-modal	Separable	–
DTLZ2	Concave	Unimodal	Separable	–
DTLZ3	Concave	Multi-modal	Separable	–
DTLZ4	Concave	Unimodal	Separable	Biased
DTLZ5	Degenerated	Unimodal	Unknown separability	–
DTLZ6	Degenerated	Unimodal	Unknown separability	Biased
DTLZ7	disconnected	Multi-modal	Separable	–
WFG1	Mixed	Unimodal	Separable	Biased
WFG2	Convex, disconnected	Multi-modal	Non-separable	–
WFG3	Linear, degenerate	Unimodal	Non-separable	–
WFG4	Concave	Multi-modal	Separable	–
WFG5	Concave	deceptive	Separable	–
WFG6	Concave	Unimodal	Non-separable	–
WFG7	Concave	Unimodal	Separable	Biased
WFG8	Concave	Unimodal	Non-separable	Biased
WFG9	Concave	Multi-modal, deceptive	Non-separable	Biased

### Performance metrics

The performance of the proposed algorithm is investigated using two performance metrics, modified Inverted Generational Distance (IGD^+^)^36^ and HyperVolume (HV)^37^.

IGD^+^ performance metric is a modified inverted generational distance metric that quantifies both convergence and diversity characteristic of an optimization algorithm. It helps to estimate the distance between the attained and true pareto fronts, lesser the IGD^+^ value indicates better the attained solutions and is calculated as given in Eq. 7.

7 $${IGD}^{+}\left(A,B\right)=\frac{{\sum }_{x\in A}d(x,B)}{\left|A\right|}$$

where, $A$ represents the reference points in true front, $B$ represents the solutions in the attained pareto front. $d(x,B)$ represents the nearest distance from $x$ to attained front solutions, and this distance calculation from $x$ to a solution $y$ in $B$ is estimated as, $d\left(x,y\right)=\sqrt{\sum_{j=1}^{n}\text{max}{({y}_{j}-{x}_{j},0)}^{2}}$ and $n$ represents the number of objectives.

HV performance metric helps to evaluate the convergence and diversity properties of the obtained solutions. HV calculates the hypervolume between the attained front and a given reference point. Formula to calculate HV is given in Eq. 8. A higher HV value indicates that better solutions are attained.

8 $$HV=Le\left({\cup }_{X\in S}\left[{f}_{1}\left(X\right),{re}_{1}\right]*\dots \left[{f}_{n}\left(X\right),{re}_{n}\right]\right)$$

where variable $S$ represents attained solution set and $Le$ indicates the Lebesgue measure. Variable $n$ denotes the number of objective functions. $Re=({re}_{1}, {re}_{2},..,{re}_{m})$ is a vector of maximum reference point value on every objective function set at (1,1). Before calculating HV metric value, the objective functions are normalized using Min–max normalization.

### Results and discussion on DTLZ problems

**Figure 2:**
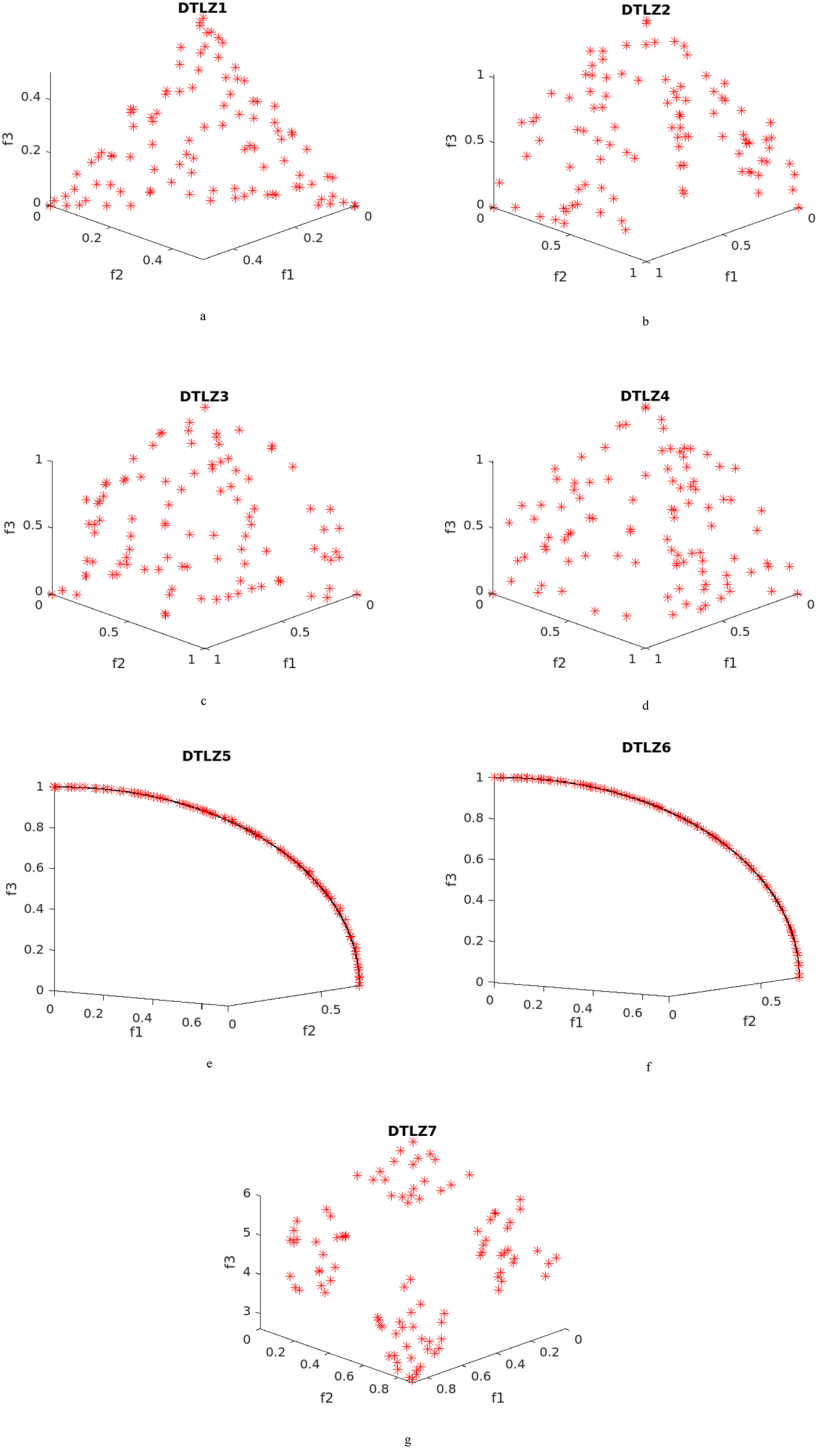
Obtained pareto fronts for DTLZ problems. (**a**) Obtained pareto fronts for DTLZ1 problem. (**b**) Obtained pareto fronts for DTLZ2 problem. (**c**) Obtained pareto fronts for DTLZ3 problem. (**d**) Obtained pareto fronts for DTLZ4 problem. (**e**) Obtained pareto fronts for DTLZ5 problem. (**f**) Obtained pareto fronts for DTLZ6 problem. (**g**) Obtained pareto fronts for DTLZ7 problem.

The mean IGD^+^ value results of DTLZ problems are given in Table 5 and the pareto fronts are given in Fig. 2. The results of HV Metric is listed in Table 6. The overall performance of NDE algorithm results is better when compared to all algorithms. DTLZ1 test problem is a linear and multi-modal test problem and chances of getting stuck at local optimal solutions are higher for problems of such characteristics, the obtained pareto front is shown in Fig. 2a. NDE algorithms performance is best followed by TSEA algorithm and the other algorithms. This is because of the fuzzy system based control parameter adaptation to regulate the population diversity and the applied denoising method. DTLZ2 is a concave, unimodal problem and the possibility of noise affecting its performance is high. NDE outperforms and the results are better compared to the other algorithms, and the respective attained front is shown in Fig. 2b. The effectiveness of averaging based denoising method is evident through the results. The characteristic of DTLZ3 test problem is multimodal as well and the performance of the proposed NDE algorithm is better, and the attained front is given in Fig. 2c.

**Table 5:** Mean IGD^+^ values for DTLZ Problems (best results are highlighted).

Problems	$\sigma$	NDE	TSEA	Two_Arch2	RMNSGAII	NTSPEA	RTEA	FNSGA-II
DTLZ1	0.1	**6.94**	7.05	12.1	25.9	20.1	16.0	8.4
	0.2	7.15	**6.79**	13.1	24.9	19.6	17.0	7.10
	0.5	**10.1**	10.4	17.7	27.2	23.9	17.6	10.6
DTLZ2	0.1	**0.0338**	0.0442	0.211	0.171	0.0969	0.0919	0.0512
	0.2	**0.0876**	0.0970	0.363	0.273	0.238	0.245	0.0891
	0.5	**0.187**	0.194	0.638	0.478	0.363	0.608	0.203
DTLZ3	0.1	**15.4**	16.8	34.9	75.2	66.8	45.2	16.5
	0.2	**18.2**	19.1	39.4	75.8	63.1	42.4	19.5
	0.5	**25.7**	26.1	41.3	70.5	68.6	47.8	25.9
DTLZ4	0.1	0.164	0.176	0.311	0.316	**0.125**	0.228	0.201
	0.2	0.321	0.341	0.474	0.476	**0.256**	0.301	0.337
	0.5	0.448	0.459	0.760	0.710	**0.378**	0.732	0.454
DTLZ5	0.1	**0.0399**	0.0485	0.195	0.191	0.0935	0.0951	0.0534
	0.2	**0.098**	0.100	0.357	0.276	0.246	0.228	0.102
	0.5	**0.188**	0.195	0.605	0.485	0.361	0.604	0.203
DTLZ6	0.1	**0.237**	0.240	1.57	2.88	0.682	1.73	0.345
	0.2	**0.428**	0.432	3.64	5.69	1.90	3.44	0.445
	0.5	**1.35**	1.37	6.45	10.7	4.16	7.17	1.45
DTLZ7	0.1	**0.821**	0.844	1.18	1.47	0.888	1.07	0.834
	0.2	**0.868**	0.872	2.15	2.93	1.44	2.11	0.885
	0.5	**0.839**	0.848	2.76	3.94	1.81	3.84	0.956

**Table 6:** Mean HV values for DTLZ Problems (best results are highlighted).

Problems	$\sigma$	NDE	TSEA	Two_Arch2	RMNSGAII	NTSPEA	RTEA	FNSGA-II
DTLZ1	0.1	**0.998**	0.992	0.978	0.906	0.942	0.962	0.897
	0.2	0.981	**0.989**	0.960	0.863	0.909	0.932	0.976
	0.5	**0.995**	0.990	0.974	0.938	0.952	0.974	0.982
DTLZ2	0.1	**0.645**	0.621	0.464	0.510	0.573	0.578	0.598
	0.2	**0.804**	0.789	0.648	0.703	0.715	0.714	0.795
	0.5	**0.851**	0.846	0.683	0.744	0.787	0.698	0.849
DTLZ3	0.1	**0.987**	0.985	0.950	0.777	0.811	0.915	0.973
	0.2	0.984	**0.990**	0.967	0.882	0.914	0.961	0.975
	0.5	**0.992**	0.979	0.957	0.878	0.878	0.940	0.986
DTLZ4	0.1	0.824	0.816	0.773	0.774	**0.832**	0.800	0.805
	0.2	0.775	0.769	0.727	0.735	0.801	0.789	**0.806**
	0.5	0.786	0.775	0.706	0.725	**0.822**	0.723	0.784
DTLZ5	0.1	**0.612**	0.598	0.453	0.467	0.555	0.553	0.594
	0.2	**0.687**	0.665	0.450	0.530	0.546	0.566	0.679
	0.5	**0.843**	0.835	0.676	0.723	0.774	0.679	0.828
DTLZ6	0.1	**0.994**	0.987	0.939	0.876	0.975	0.934	0.945
	0.2	**0.992**	0.986	0.859	0.708	0.942	0.869	0.889
	0.5	**0.987**	0.984	0.863	0.680	0.934	0.847	0.975
DTLZ7	0.1	**0.624**	0.597	0.548	0.507	0.591	0.567	0.594
	0.2	**0.716**	0.706	0.573	0.492	0.652	0.600	0.708
	0.5	**0.719**	0.700	0.507	0.422	0.608	0.433	0.693

**Figure 3:**
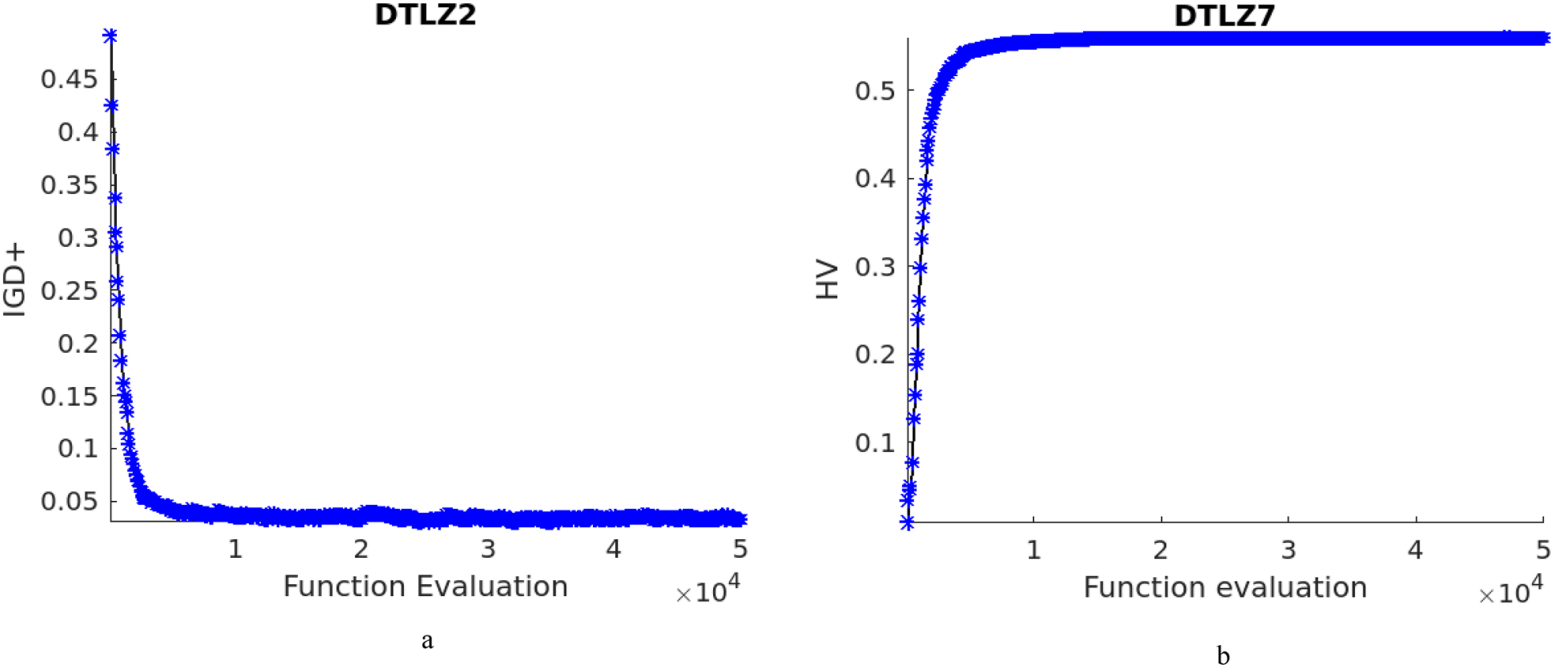
(**a**) IGD + values across the function evaluations for DTLZ2 test problem. (**b**) HV values across the function evaluations for DTLZ7 test problem.

DTLZ4 test problem is a modified one from DTLZ2 and the solutions are usually densely populated near to the planes. The performance of NTSPEA algorithm is better followed by NDE and the other methods. It can be observed that the NTSPEA algorithm works by associating survival time factor for all the solutions in the population and DTLZ4 problem is sensitive towards the initial population, and the respective attained front is given in Fig. 2d. The characteristics of test problems DTLZ5, DTLZ6 and DTLZ7 are irregular and the pareto front for DTLZ7 is discontinuous, the obtained pareto fronts for these problems are illustrated in Fig. 2e, f and g respectively. The proposed NDE algorithm attains better results for all the above problems. The IGD^+^ and HV values evolution across the function evaluations for DTLZ2 and DTLZ7 test problems are given in Fig. 3a and b respectively.

### Results and discussion on WFG problems

**Figure 4:**
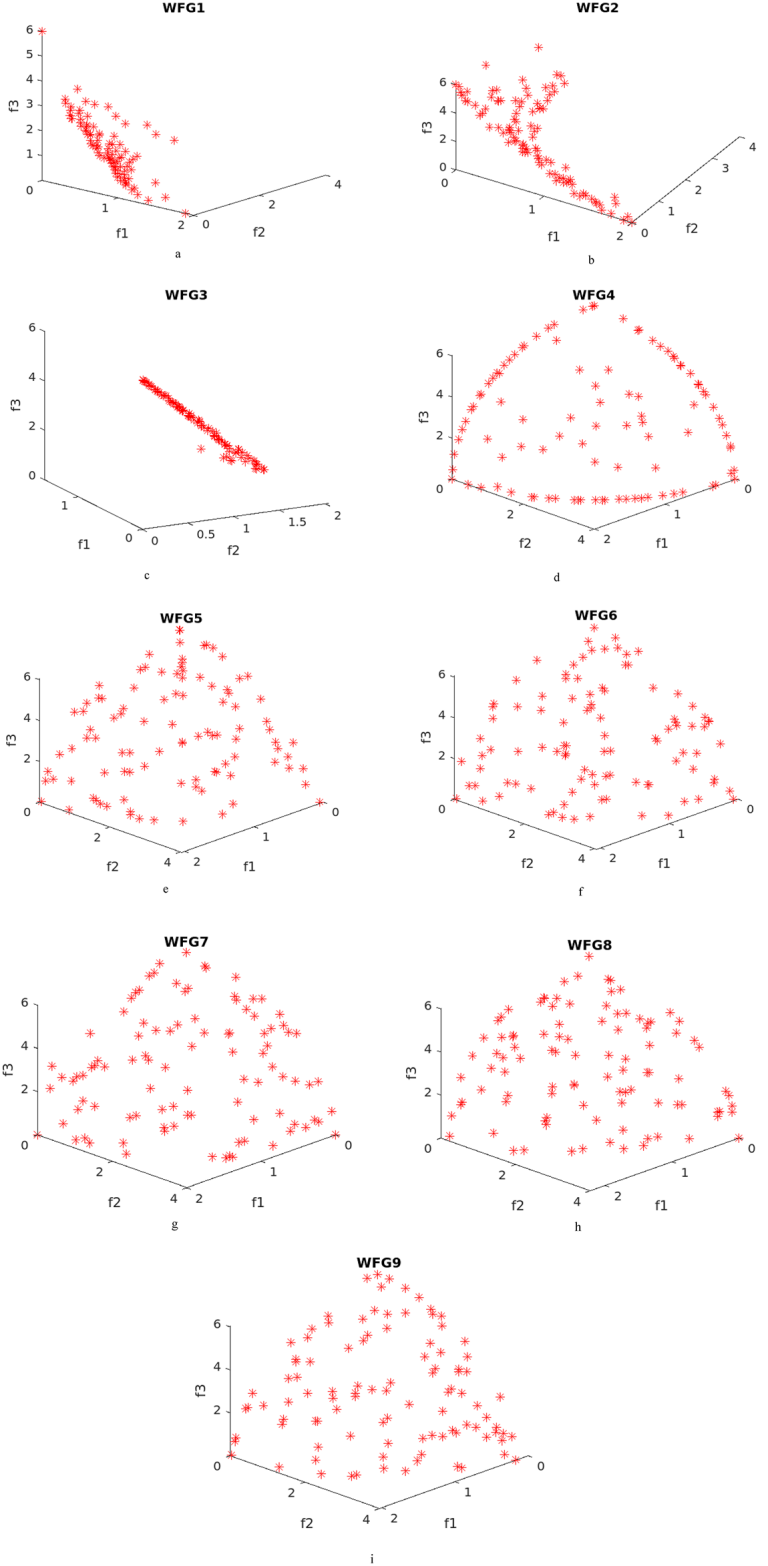
Obtained pareto fronts for WFG problems. (**a**) Obtained pareto fronts for WFG1 problem. (**b**) Obtained pareto fronts for WFG2 problem. (**c**) Obtained pareto fronts for WFG3 problem. (**d**) Obtained pareto fronts for WFG4 problem. (**e**) Obtained pareto fronts for WFG5 problem. (**f**) Obtained pareto fronts for WFG6 problem. (**g**) Obtained pareto fronts for WFG7 problem. (**h**) Obtained pareto fronts for WFG8 problem. (**i**) Obtained pareto fronts for WFG9 problem.

WFG test problems are relatively complex when compared to the DTLZ problems. The mean values of the IGD^+^ and HV performance metrics are given in Tables 7, 8 respectively. The attained pareto fronts for the WFG problems are given in Fig. 4. WFG1 test problem is a complex test problem with mixed and biased characteristics. The performance of the proposed NDE algorithm is better than other algorithms taken for comparison, and is at par with performance of NTSPEA algorithm, the obtained pareto front for the same is illustrated in Fig. 4a. WFG2 is a multimodal test problem, and the performance of NDE is the best, and the respective front is given in Fig. 4b. WFG3 is a linear, non-separable test problem and the attained solutions and the mean IGD^+^ and HV values are better than other methods, and the pareto front is given in Fig. 4c.

**Table 7:** Mean IGD^+^ values for WFG problems (best results are highlighted).

Problems	$\sigma$	NDE	TSEA	Two_Arch2	RMNSGAII	NTSPEA	RTEA	FNSGA-II
WFG1	0.1	**1.14**	1.28	1.31	1.30	1.29	1.29	1.25
	0.2	**0.741**	1.30	0.824	0.777	0.775	0.820	0.98
	0.5	**1.37**	1.56	1.77	1.73	1.42	1.60	1.54
WFG2	0.1	**0.118**	0.142	0.249	0.250	0.189	0.221	0.124
	0.2	**0.204**	0.216	0.359	0.334	0.307	0.304	0.219
	0.5	**0.337**	0.447	0.579	0.468	0.455	0.416	0.400
WFG3	0.1	**0.138**	0.157	0.278	0.260	0.197	0.206	0.165
	0.2	**0.221**	0.239	0.457	0.337	0.356	0.305	0.258
	0.5	**0.467**	0.542	0.676	0.492	0.544	0.471	0.497
WFG4	0.1	**0.101**	0.105	0.177	0.160	0.127	0.113	0.112
	0.2	**0.149**	0.157	0.279	0.222	0.226	0.174	0.164
	0.5	**0.291**	0.327	0.470	0.336	0.352	0.296	0.318
WFG5	0.1	**0.120**	0.129	0.243	0.260	0.161	0.177	0.130
	0.2	**0.186**	0.190	0.385	0.414	0.298	0.271	0.214
	0.5	**0.337**	0.443	0.630	0.605	0.467	0.453	0.460
WFG6	0.1	**0.138**	0.148	0.264	0.244	0.179	0.160	0.152
	0.2	**0.204**	0.216	0.412	0.366	0.328	0.269	0.211
	0.5	0.498	0.525	0.691	0.592	0.554	**0.470**	0.493
WFG7	0.1	**0.0787**	0.0894	0.195	0.192	0.122	0.138	0.110
	0.2	**0.140**	0.151	0.351	0.302	0.272	0.220	0.154
	0.5	**0.346**	0.377	0.575	0.450	0.436	0.403	0.365
WFG8	0.1	**0.192**	0.194	0.285	0.291	0.217	0.235	0.196
	0.2	**0.237**	0.248	0.410	0.360	0.348	0.306	0.254
	0.5	**0.460**	0.563	0.630	0.496	0.489	0.463	0.578
WFG9	0.1	**0.102**	0.104	0.184	0.182	0.124	0.144	0.114
	0.2	**0.158**	0.161	0.247	0.253	0.215	0.219	0.169
	0.5	**0.251**	0.266	0.558	0.409	0.359	0.363	0.319

**Table 8:** Mean HV values for WFG problems (best results are highlighted).

Problems	$\sigma$	NDE	TSEA	Two_Arch2	RMNSGAII	NTSPEA	RTEA	FNSGA-II
WFG1	0.1	**0.415**	0.404	0.383	0.390	0.401	0.400	0.409
	0.2	**0.565**	0.391	0.538	0.559	0.563	0.545	0.384
	0.5	0.297	0.283	0.203	0.217	**0.335**	0.267	0.276
WFG2	0.1	**0.624**	0.619	0.558	0.559	0.591	0.576	0.595
	0.2	**0.610**	0.604	0.530	0.545	0.556	0.560	0.587
	0.5	**0.541**	0.517	0.459	0.510	0.519	0.535	0.508
WFG3	0.1	**0.582**	0.579	0.515	0.526	0.557	0.556	0.564
	0.2	**0.587**	0.570	0.462	0.522	0.512	0.540	0.578
	0.5	**0.510**	0.472	0.406	0.490	0.471	0.498	0.502
WFG4	0.1	**0.368**	0.362	0.319	0.331	0.349	0.357	0.349
	0.2	**0.374**	0.371	0.300	0.335	0.333	0.362	0.365
	0.5	**0.325**	0.305	0.209	0.293	0.283	0.322	0.312
WFG5	0.1	**0.392**	0.384	0.320	0.315	0.366	0.360	0.375
	0.2	**0.407**	0.406	0.305	0.298	0.351	0.370	0.397
	0.5	**0.318**	0.312	0.204	0.231	0.293	0.311	0.301
WFG6	0.1	**0.415**	0.390	0.327	0.340	0.373	0.384	0.380
	0.2	**0.406**	0.404	0.301	0.331	0.346	0.379	0.397
	0.5	0.330	0.306	0.215	0.275	0.283	**0.333**	0.324
WFG7	0.1	**0.409**	0.407	0.349	0.353	0.389	0.382	0.397
	0.2	**0.421**	0.410	0.303	0.335	0.346	0.376	0.410
	0.5	**0.356**	0.341	0.230	0.306	0.310	0.334	0.346
WFG8	0.1	**0.378**	0.370	0.319	0.320	0.356	0.351	0.368
	0.2	**0.394**	0.377	0.290	0.323	0.325	0.351	0.385
	0.5	**0.361**	0.307	0.260	0.337	0.335	0.355	0.314
WFG9	0.1	**0.419**	0.407	0.364	0.368	0.396	0.388	0.400
	0.2	**0.402**	0.394	0.346	0.349	0.366	0.368	0.386
	0.5	**0.417**	0.409	0.255	0.339	0.363	0.370	0.413

**Figure 5:**
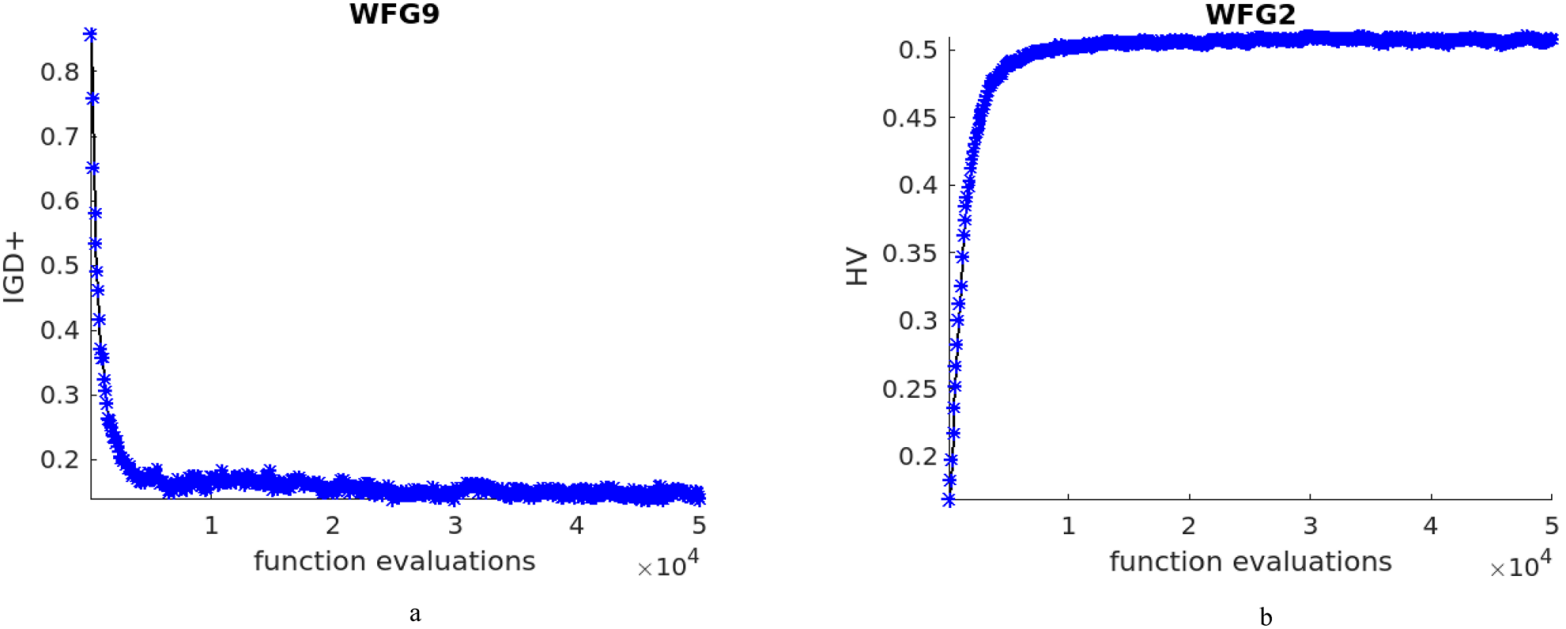
(**a**) IGD + values across the function evaluations for WFG9 test problem. (**b**) HV values across the function evaluations for WFG2 test problem.

The characteristic of WFG4 problem is that it is multi-modal, with multiple local optimal solutions. The population diversity control and the appropriate denoising method aid in escaping from such locally optimal solutions and it is evident through the obtained results, and front as given in Fig. 4d. WFG5 problem is of deceptive characteristic and is a separable problem and the attained concave pareto front and the mean IGD^+^ and HV values are better, and the front for the problem is illustrated in Fig. 4e. WFG6, WFG8 and WFG9 test problems are non-separable test problems and WFG9 is multi-modal as well. For the test problem WFG6, the performance of NDE and RTEA are at par, followed by the other algorithms, and the attained pareto front is illustrated in Fig. 4f. For WFG8 and WFG9 test problems, the results of NDE algorithm are promising, and the obtained pareto fronts for these problems are given in Fig. 4h and i respectively. The characteristic of WFG7 is it is unimodal and a separable one and the results of the proposed NDE algorithm is best, and the pareto front is given in Fig. 4g. The IGD^+^ and HV values evolution across the function evaluations for WFG9 and WFG2 test problems are given in Fig. 5a and b respectively.

### Population effect and population diversity analysis

Population plays a vital role in search and optimization algorithms. The initial population is randomly generated within the variable bounds. Along the evolution, fitter solutions are selected and used that helps to attain an optimal or pareto-optimal solutions. In the proposed research, the population diversity plays a key role. The population diversity is controlled adaptively in order to improve the search performance. The solutions in the population must be diverse enough during the initial stages of evolution to have a better exploration over the search space and over the evolution and at later stages the population diversity is to be low to have better exploitation. Population that aids in balancing the explore-exploit cycle is required to attain optimal or pareto optimal solutions. The effect of population diversity in the present research is presented below and the explore-exploit cycle analysis is presented in the section "Exploration–Exploitation analysis".

To perform population diversity analysis, diversity of the population is calculated as given in Eq. 9, “distance to average point” measure^32^. This measure includes population size, dimension and search region of variables, thus this measure used for population diversity estimation.

9 $${diversity}_{generation}\left(PP\right)= \frac{1}{|LenDia|*NP}*{\sum }_{i=1}^{NP}\sqrt{{\sum }_{j=1}^{{d}_{i}}({y}_{ij}-{\overline{y}}_{j}}{)}^{2}$$

where, $PP$ represents population with size $NP$. $\left|LenDia\right|$ is the diagonal length of the search space, ${d}_{i}$ is the problem dimension. ${y}_{ij}$ is ${j}^{th}$ value of ${i}^{th}$ solution, ${\overline{y}}_{j}$ is the ${j}^{th}$ value of average point $\overline{y }$.

**Figure 6:**
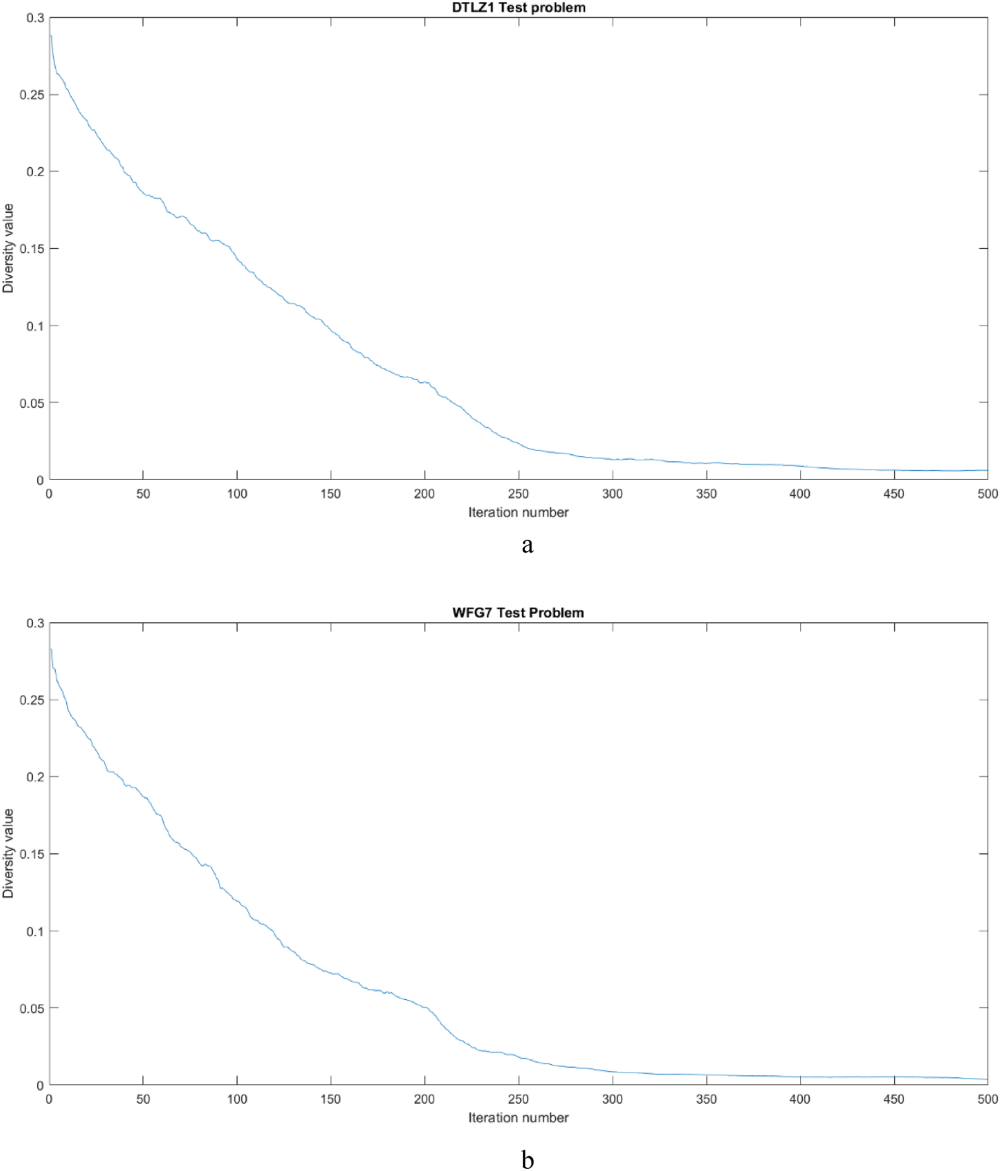
(**a**) Population diversity adaptation along the iterations for DTLZ1 test problem. (**b**) Population diversity adaptation along the iterations for WFG7 test problem.

Figure 6a and b shows the diversity graph of the population along the iterations for two problems, from which it is evident that, during the initial stages of evolution higher diversity is observed to improve the exploration and in later stage diversity value is lesser to improve exploitation.

### Exploration–Exploitation analysis

To analyse the balancing property of exploration–exploitation cycle, the exploration and exploitation rate along the evolution is calculated using Eqs. 10 and 11^40^.

10 $$exploration\;\left(iteration\right)=\left(\frac{popdiversity(iteration)}{\text{max}(popdiversity(iteration)}\right)\times 100$$

11 $$exploitation\;\left(iteration\right)=\left(\frac{|popdiversity\left(iteration\right)-\text{max}(popdiversity(iteration)|}{\text{max}(popdiversity\left(iteration\right))}\right)\times 100$$

where, $popdiversity$ indicates the population diversity and is calculated using Eq. 9.

**Figure 7:**
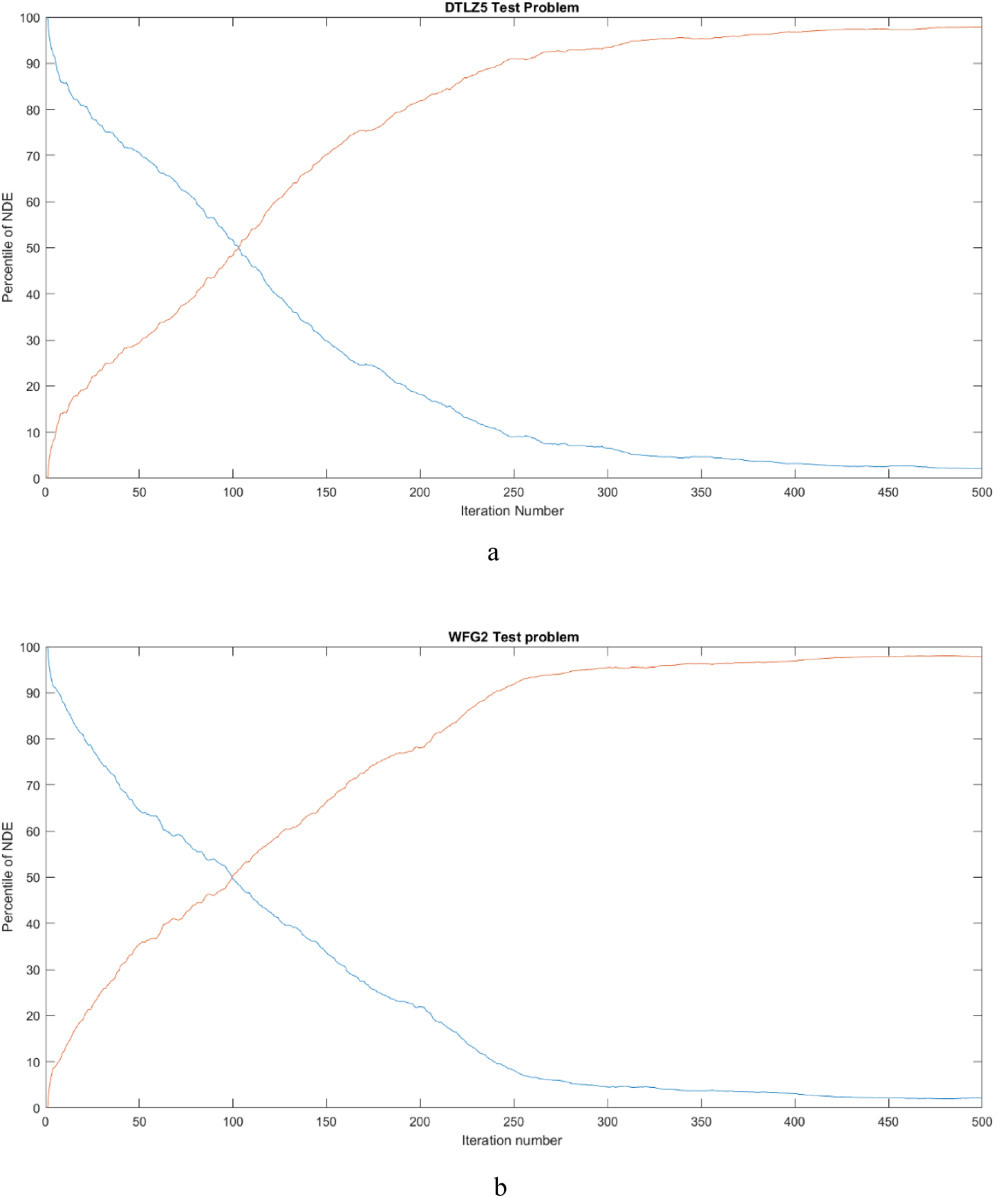
(**a**) Exploration–exploitation graph for DTLZ5 test problem. (**b**) Exploration–exploitation graph for WFG2 test problem.

Exploration–exploitation graph for two problems is given in Fig. 7a and b. It can be observed that, exploration starts with a higher value and during later stages it decreases and the exploitation begins with a smaller value and upon evolution its value is increased in final stages, which ensures the balance between the cycles of exploration and exploitation.

### Statistical test results

Statistical analysis through Wilcoxon signed rank test and Friedman test^41^ are performed to further validate the performance of NDE.

Wilcoxon signed rank test is a pairwise comparison test, used to find whether there is significant difference between two algorithms. The test is performed using IBM SPSS package and the significance level $\alpha$ is set to 0.05. $\rho$-value is computed through the above test and if the obtained $\rho$-value is less than $\alpha$, the null hypothesis can be rejected and it implies there is significance difference among the algorithms taken for evaluation. The test results using the mean HV value results with $\sigma$ value 0.5 obtained through various algorithms for DTLZ problems is presented in Table 9. The $\rho$-value is less than 0.05 (significance level) and the null hypothesis can be rejected. It is evident that the proposed NDE algorithm is significantly better when compared to other algorithms taken for comparison.

**Table 9:** Wilcoxon signed rank test results for mean HV values against DTLZ problems.

NDE versus	R^+^	R^-^	Test statistic value	$\rho$-value
TSEA	7	0	− 2.371	0.018
Two_Arch2	7	0	− 2.366	0.018
RMNSGAII	7	0	− 2.366	0.018
NTSPEA	6	1	− 2.197	0.028
RTEA	7	0	− 2.366	0.018
FNSGA-II	7	0	− 2.371	0.018

Table 10 gives the Wilcoxon signed rank test results using the mean IGD^+^ value results with.

**Table 10:** Wilcoxon signed rank test results for mean IGD^+^ values against WFG problems.

NDE vs	R^+^	R^-^	Test statistic value	$\rho$-value
TSEA	9	0	− 2.666	0.008
Two_Arch2	9	0	− 2.666	0.008
RMNSGAII	9	0	− 2.666	0.008
NTSPEA	9	0	− 2.666	0.008
RTEA	8	1	− 2.192	0.028
FNSGA-II	8	1	− 2.547	0.011

$\sigma$ value 0.5 obtained through various algorithms for WFG test problems. It is evident that the NDE algorithm is significantly better than the other algorithms taken for comparison as the $\rho$-value is less than 0.05.

The competence of the proposed NDE algorithm is also statistically analysed using Friedman rank test, which is a multiple comparison test. Joint analysis of algorithms taken for comparison is performed through the above test. The test is performed using IBM SPSS package and the significance level $\alpha$ is set to 0.05. $\rho$-value is computed through the above test and if the obtained $\rho$-value is less than $\alpha$, it implies there is significance difference among the algorithms taken for evaluation.

Table 11 gives the Friedman rank test results using the mean IGD^+^ value results with.

**Table 11:** Friedman rank test results for mean IGD^+^ values against DTLZ problems.

Problems	NDE	TSEA	Two_Arch2	RMNSGAII	NTSPEA	RTEA	FNSGA-II
DTLZ1	1	2	5	7	6	4	3
DTLZ2	1	2	7	5	4	6	3
DTLZ3	1	3	4	7	6	5	2
DTLZ4	2	4	7	5	1	6	3
DTLZ5	1	2	7	5	4	6	3
DTLZ6	1	2	5	7	4	6	3
DTLZ7	1	2	5	7	4	6	3
Friedman value	1.14	2.43	5.71	6.14	4.14	5.57	2.86
Overall rank	1	2	6	7	4	5	3

$\sigma$ value 0.5 obtained through various algorithms for DTLZ problems.

The proposed NDE algorithm is ranked first in the above test and the $\rho$-value obtained is 0.0001 which is less than the significance level $\alpha =0.05$, which proves the significance of the proposed optimization algorithm.

Table 12 gives the Friedman rank test results using the mean HV value results with $\sigma$ value 0.5 obtained through various algorithms for WFG problems.

**Table 12:** Friedman rank test results for mean HV values against WFG problems.

Problems	NDE	TSEA	Two_Arch2	RMNSGAII	NTSPEA	RTEA	FNSGA-II
WFG1	2	3	7	6	1	5	4
WFG2	1	4	7	5	3	2	6
WFG3	1	5	7	4	6	3	2
WFG4	1	4	7	5	6	2	3
WFG5	1	2	7	6	5	3	4
WFG6	2	4	7	6	5	1	3
WFG7	1	3	7	6	5	4	2
WFG8	1	6	7	3	4	2	5
WFG9	1	3	7	6	5	4	2
Friedman value	1.22	3.78	7.00	5.22	4.44	2.89	3.44
Overall rank	1	4	7	6	5	2	3

The proposed NDE algorithm is again ranked first in the above statistical test and the $\rho$-value obtained is 0.00008 which is less than the significance level $\alpha =0.05$, shows the significance of the proposed optimization algorithm.

### Experimentation on CEC 2017 test problems

To further investigate the effectiveness of the proposed Differential Evolution based Noise handling Optimization algorithm (NDE), experimentation is conducted on CEC 2017 test problems^42^ which are single objective optimization problems. To perform this study, the selection process in the proposed NDE algorithm is changed suitable to handle single objective optimization problems. The trial vector generated after the crossover operation is subject to fitness function evaluation. The fitness function values of the solutions in target and trial vectors are compared and the better solution is chosen as candidate for the next generation.

CEC 2017 test suite comprises 29 test problems (Test problem F2 has been removed from the CEC 2017 suite). The parameter settings are followed as given in the technical report and are given in Table 13.

**Table 13:** Parameter settings for CEC 2017 test problem evaluation.

Parameter	Value
Dimension $(D)$	50
Runs	51
Maximum number of function evaluations	$D*10000$
Search range	${[-\text{100,100}]}^{D}$

The result analysis is performed by calculating error value, which is the difference between the best objective function value that is attained in a run and the true optimal value. This error value is calculated for all the 51 runs and the mean and standard deviation values are recorded. The results are compared with two other algorithms Effective Butterfly Optimizer using Covariance Matrix Adapted Retreat phase (EBOwithCMAR)^43^ and jSO^44^ which has secured first and second rank in the CEC 2017 competition and are presented in Table 14. The best results are highlighted in boldface, and it can be observed that the proposed NDE algorithm performs better in 19 functions, similar performance in 3 functions and inferior performance in 7 functions. This shows the significance of the NDE algorithm, with its capability of population diversity control through strategy adaptations, the restricted local search and noise handling techniques exhibits a robust performance.

**Table 14:** Mean and standard deviation results of the error values.

Test function	NDE	EBOwithCMAR	jSO
F1	0 ± 0	0 ± 0	0 ± 0
F3	0 ± 0	0 ± 0	0 ± 0
F4	**39.4 ± 28.72**	42.9 ± 33.2	56.213 ± 48.763
F5	**7.24 ± 2.321**	7.58 ± 2.42	16.405 ± 3.4620
F6	0.000000453 ± 0.000000129	**0.0000000854 ± 0.000000114**	0.00000109 ± 0.00000262
F7	**53.39 ± 1.29**	57.90 ± 1.53	66.497 ± 3.4728
F8	**7.74 ± 2.75**	7.91 ± 2.47	16.962 ± 3.1354
F9	0 ± 0	0 ± 0	0 ± 0
F10	**2976 ± 412**	3110 ± 401	3139.8 ± 367.16
F11	**24.87 ± 3.24**	26.40 ± 3.36	27.939 ± 3.3284
F12	**1636 ± 674.12**	1940 ± 834	1680.6 ± 522.93
F13	36.23 ± 23.1	41.4 ± 24.8	**30.599 ± 21.226**
F14	26.162 ± 2.455	31.2 ± 3.52	**24.964 ± 1.8734**
F15	25.12 ± 3.96	29.4 ± 5.20	**23.864 ± 2.4882**
F16	**310 ± 127**	346 ± 146	450.52 ± 137.75
F17	**272 ± 87.12**	275 ± 86.3	282.87 ± 86.142
F18	**23.1 ± 4.321**	32.0 ± 5.99	24.283 ± 2.0174
F19	**12.2 ± 3.578**	24.5 ± 3.94	14.139 ± 2.2622
F20	**135 ± 76.18**	147 ± 74.4	140.10 ± 77.375
F21	**207 ± 3.866**	211 ± 4.06	219.20 ± 3.7656
F22	793.1 ± 1134.8	**365 ± 924**	1487.2 ± 1753.1
F23	**427 ± 7.65**	434 ± 8.16	430.08 ± 6.2364
F24	**496 ± 3.54**	506 ± 3.85	507.45 ± 4.1273
F25	**472 ± 8.544**	489 ± 24.7	480.88 ± 2.7999
F26	951 ± 89.554	**706 ± 406**	1128.8 ± 56.167
F27	**492 ± 8.456**	522 ± 7.75	511.27 ± 11.077
F28	**452.1 ± 7.45**	467 ± 17.9	459.81 ± 6.8398
F29	384 ± 17.66	**347 ± 19.7**	362.94 ± 13.157
F30	**600,683 ± 32,567**	618,000 ± 36,200	601,050 ± 29,859

### Limitations

The main limitations of the research include, experimenting on a number of benchmark problems with varied and complex characteristics and not conducting experimentation on a real-world optimization problem^45–47^. The widely considered limitation in applying an optimization algorithm to a real-world problem will be parameter fine tuning according to the problem. But in the proposed NDE algorithm the major parameters like crossover rate and trial vector generation strategies are self-adapted according to the population domain of the problem. Thus, finetuning the other associated parameters may be a challenge. Through the above test results the robustness of the proposed NDE algorithm in solving a wider range of problems is evident. Further improvements will be studied in future work, by integrating multiple denoising models, fine tuning algorithm to handle constrained optimization problems and conducting experiments on real-world optimization problem.

## Conclusions

In the present work, Differential Evolution based Noise handling Optimization algorithm (NDE) to optimize noisy bi-objective optimization problems is proposed. FAMDE-DC is used as the base optimizer, where fuzzy system is extended to adapt crossover rate in order to control the population diversity. Adaptive switching technique is extended to test whether the noise ratio is within the acceptable limits or not. If the noise ratio is exceeding the prespecified limits, then explicit averaging based denoising method is applied. To further improve the convergence characteristics, a restricted local search procedure is applied in prespecified intervals. To evaluate the performance of proposed NDE algorithm, DTLZ and WFG test problems are used by including noise of different levels. The modified Inverted Generational Distance (IGD^+^) and Hypervolume (HV) are chosen as the performance indicators. The attained results for most of the above problems using NDE algorithm are better than SOTA algorithms that are taken for comparison and shows the effectiveness of NDE algorithm in handling noisy optimization problems. The results related to test problem DTLZ4 are not best, the pareto optimal solutions for the problem are densely populated near to the planes and the convergence characteristics of the attained solutions are to be improved, which shows the future direction of improving the NDE algorithm. Further, non-parametric statistical tests namely Wilcoxon signed rank test and Friedman rank tests are conducted on the attained results, and the test results shows the significance of the suggested NDE algorithm over the other algorithms taken for comparison.

## Data Availability

The datasets used and analysed during the current study available from the corresponding author on reasonable request.
